# Sequential allogeneic HSCT after CAR-T therapy for relapsed/refractory acute lymphoblastic leukemia patients: A long-term follow-up result

**DOI:** 10.1016/j.jare.2025.02.006

**Published:** 2025-02-11

**Authors:** Tingting Yang, Yetian Dong, Jimin Shi, Mingming Zhang, Delin Kong, Jingjing Feng, Shan Fu, Pingnan Xiao, Ruimin Hong, Huijun Xu, Yi Luo, Yanmin Zhao, Jian Yu, Xiaoyu Lai, Lizhen Liu, Huarui Fu, Yishan Ye, Dawei Cui, Jiazhen Cui, Simao Huang, Guoqing Wei, Alex H. Chang, He Huang, Yongxian Hu

**Affiliations:** a Bone Marrow Transplantation Center of The First Affiliated Hospital Liangzhu Laboratory, Zhejiang University School of Medicine, Hangzhou, Zhejiang, China; b Institute of Hematology, Zhejiang University, Hangzhou, Zhejiang, China; c Zhejiang Province Engineering Laboratory for Stem Cell and Immunity Therapy, Hangzhou, Zhejiang, China; d Department of Blood Transfusion, The First Affiliated Hospital, Zhejiang University School of Medicine, Hangzhou, Zhejiang, China; e Engineering Research Center of Gene Technology, Ministry of Education, Institute of Genetics, School of Life Sciences, Fudan University, Shanghai, China; f Shanghai YaKe Biotechnology Ltd., Shanghai, China

**Keywords:** R/R B-ALL, CAR-T, HSCT, Long-term follow-up

## Abstract

**Introduction:**

CAR-T cell therapy has revolutionized the therapeutic landscape for relapsed/refractory (R/R) B-cell acute lymphoblastic leukemia (B-ALL), and bridging with allogeneic hematopoietic stem cell transplantation (allo-HSCT) has the potential to lower relapse rates. Nevertheless, the majority of existing studies have exclusively focused on short-term outcomes, resulting in a lack of comprehensive understanding of the long-term sustainability of overall prognosis.

**Objectives:**

Our study aimed to provide real-world, long-term follow-up data for patients who underwent sequential therapy.

**Methods:**

Patients with R/R B-ALL who achieved MRD^-^CR following CAR-T therapy and subsequently underwent allo-HSCT between January 2016 and May 2024 were enrolled. The primary outcomes included overall survival (OS), leukemia-free survival (LFS), non-relapse mortality (NRM) and cumulative incidence of relapse (CIR). Acute and chronic graft-versus-host disease (GVHD) and graft-versus-host disease-free survival (GRFS) were also investigated.

**Results:**

The median age at transplant of 32.1 years. Of these patients, 88.2 % underwent haploidentical-HSCT, and 11.8 % received either unrelated matched or related matched HSCT. The cumulative incidences of grades I-IV and grade II-IV aGVHD at day 100 were 31.4 % and 15.7 %, respectively. The cumulative incidence of cGVHD at 4 years was 48.3 %. With a median follow-up time of 43.2 months, OS, LFS, and GRFS at 4 years were 68.9 %, 61.4 %, and 39.5 %, respectively. Fifteen cases (29.4 %) experienced relapse, predominantly antigen-positive relapse (n = 11). NRM and CIR at 4 years were 10.6 % and 28.0 %, respectively. In the multivariate analyses, patients over 45 years of age and with poor-risk had significantly dismal OS (*P* = 0.018; *P* = 0.038) and LFS (*P* = 0.01; *P* = 0.03).

**Conclusion:**

Our study exhibits favorable long-term outcomes consistent with those reported in clinical trials, with sustained, durable responses observed at the 4-year follow-up. However, these benefits are less pronounced in older patients and those with poor-risk disease characteristics.

## Highlights

• This study provides the longest follow-up real-world data on sequential allo-HSCT post-CAR-T therapy.
• Sequential allo-HSCT after CAR-T treatment shows durable remissions in patients achieving MRD-negative CR.
• With 4 years of follow-up, overall survival reaches 68.9 %, highlighting the long-term benefits.
• Sequential therapy demonstrates manageable safety, with an aGVHD incidence of 31.4% and no GVHD-related deaths.
• Age and high-risk genetic factors are key determinants of long-term outcomes, requiring personalized treatment strategies.

## Graphical abstract

**Figure:**
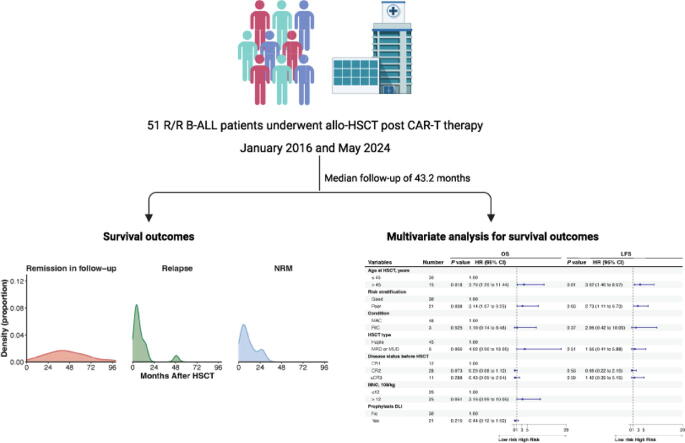

## Introduction

Relapsed/refractory (R/R) B-cell acute lymphoblastic leukemia (B-ALL) represents one of the most challenging scenarios in hematological malignancies. Historically, the prognosis for adult patients with R/R B-ALL has been poor, with limited treatment options available [1]. The advent of chimeric antigen receptor-T (CAR-T) cell therapy has heralded a new era in the treatment of R/R B-ALL, offering a potentially curative option for patients who previously had few alternatives. CAR-T therapy involves the genetic modification of autologous or allogeneic T cells to express synthetic receptors capable of recognizing tumor-associated antigens, most notably CD19 and CD22, which are widely expressed on malignant B cells. Upon reinfusion, these engineered CAR-T cells actively eliminate leukemic cells through potent cytotoxic mechanisms. This therapeutic approach has demonstrated unprecedented efficacy in R/R B-ALL, achieving complete remission (CR) rates exceeding 90 % in R/R patients, and is recommended for those who fail to respond to standard chemotherapy [2], [3]. Nevertheless, the duration of response remains a significant concern, with relapse after CAR-T therapy occurring in 20–45 % of responders. Relapse manifests as either antigen-positive or antigen-negative relapse, driven by mechanisms such as antigen escape, antigen alternative splicing, lineage switching, or insufficient CAR-T cell persistence [4], [5]. These challenges underscore the need for consolidation strategies to maintain remission and prevent relapse.

Allogeneic hematopoietic stem cell transplantation (allo-HSCT), a cornerstone of curative modalities for decades, is often recommended as a consolidative approach following CAR-T therapy. Allo-HSCT provides a graft-versus-leukemia effect that donor-derived immune cells can contribute to the eradication of residual leukemic clones, thereby sustaining remission in high-risk patients. Evidence from both our previous study and others suggests that patients with minimal residual disease (MRD)-positive status are at a higher risk of relapse and can benefit greatly from bridging to allo-HSCT [6], [7], [8]. However, the role of successive consolidation with allo-HSCT in the setting of MRD-negative (MRD^-^) CR following CAR-T therapy remains a subject of considerable debate. While some studies advocate for consolidative allo-HSCT with superior overall survival (OS) and leukemia-free survival (LFS) compared to CAR-T therapy alone, others argue that the benefits may be outweighed by transplant-associated non-relapse mortality (NRM) [4], [5], [6], [7]. Furthermore, the majority of these studies are limited by short-term follow-up durations, often not extending beyond one or two years post-HSCT.

In light of these considerations, we conducted a retrospective analysis to evaluate the long-term efficacy and safety of sequential consolidation with allo-HSCT in patients who achieved MRD^-^CR following CAR-T therapy, offering insights into the potential benefits and risks of this sequential consolidation strategy over extended follow-up periods.

## Methods

### Study design

This retrospective investigation included 51 consecutive patients who underwent allo-HSCT at our center after achieving MRD^-^CR following CAR-T therapy between January 2016 and May 2024. Patients who met the following criteria were included in this study: (1) diagnosed with B-ALL, (2) underwent allo-HSCT at our hospital, regardless of previous transplantation history, and (3) achieved MRD^-^CR status following CAR-T therapy. The study was approved by the Ethics Review Committee of the First Affiliated Hospital of Zhejiang University and conducted in compliance with the Declaration of Helsinki. Written informed consent was obtained from all participants or their families.

### CAR-T protocols

As previously described, the manufacturing of CAR-T products adhered to the established protocols [9], [10], [11]. The CAR used in this study is a second-generation CAR structure, incorporating CD3ζ and 4-1BB costimulatory domains. The selection of the specific CAR-T cell targets, including CD19 or CD22, was based on the fluorescent intensity of antigen expression in leukemia cells, which was quantitatively assessed by flow cytometry. These CAR-T therapies were investigated in clinical trials registered under NCT04532281, NCT04532268, NCT04227015, ChiCTR1800015575, and ChiCTR1800017402. CAR-T cell levels were regularly monitored using flow cytometry until transplantation. Lymphodepleting chemotherapy with fludarabine and cyclophosphamide was administered before infusion, followed by CAR-T cell infusion on day 0. In this study, the CAR-T cell infusion refers to the last CAR-T infusion administered prior to the subsequent HSCT.

### Transplantation procedure and GVHD prophylaxis

The transplant procedure and graft-versus-host disease (GVHD) prophylaxis regimens were carried out following standard protocols as previously described [12], [13]. Patients underwent allo-HSCT from matched sibling donors (MSD), unrelated donors (URD), or haploidentical donors, depending on donor availability, patient-specific factors, and institutional preferences. The conditioning regimen was tailored based on the patient's disease status, age, and overall health, employing either myeloablative conditioning (MAC) or reduced-intensity conditioning (RIC). MAC regimens typically involved cytarabine from days –10 to –9, busulfan from days –8 to –6, cyclophosphamide from days –5 to –4, and methyl-N-(2-chloroethyl)-N-cyclohexyl-N-nitrosourea on day –3. For patients receiving haploidentical HSCT (haplo-HSCT), anti-thymocyte globulin (ATG) was administered as either ATG-F (Grafalon) or ATG-G (Genzyme) from days –5 to –2. URD-HSCT patients received ATG-G from days –5 or –4 to –2. The RIC regimen consisted of fludarabine from days –10 to –5, busulfan from days –5 to –6, and ATG-F (Grafalon) from days –4 to –1.

Peripheral blood stem cells were mobilized from donors with subcutaneous injections of recombinant human granulocyte colony-stimulating factor at 5 to 10  μg/kg/day for 4 to 5 consecutive days. These mobilized stem cells were harvested and then infused on days –1 and 0. GVHD prophylaxis included cyclosporine A, short-course methotrexate, and low-dose mycophenolate mofetil. Additionally, patients at high-risk of relapse were given prophylactic donor lymphocyte infusion (DLI) as a relapse prevention strategy.

### Outcome measurements

Patients were classified as either good or poor risk on the basis of the guidelines of the National Comprehensive Cancer Network (NCCN) (version 1, 2021). Poor risk was characterized by the presence of adverse cytogenetic abnormalities including hypodiploidy, t(4;11), or t(1;19), other KMT2A rearrangements, BCR-ABL1–like or Ph-like ALL, complex karyotype, and iAMP21. CR was defined as less than 5 % blasts in the bone marrow (BM), no circulating blasts, and no evidence of extramedullary disease (EMD) involvement. MRD negativity was established as less than 0.01 % BM blasts detected by flow cytometry. Leukemia relapse referred to the recurrence of ≥5 % leukemic blasts, whether in peripheral blood, BM, or the development of EMD infiltration after CR. B-cell aplasia (BCA) was defined as B lymphocytes comprising less than 3 % of lymphocytes in peripheral blood and BM.

The primary endpoints were OS, LFS, NRM, and cumulative incidence of relapse (CIR). OS was defined as the duration from transplantation to the time of death from any cause. LFS was calculated from transplantation until either relapse, death, or the last available follow-up. NRM was assessed as death resulting from causes other than leukemia relapse, and CIR was defined as the incidence of leukemia relapse following allo-HSCT. Secondary outcomes included engraftment, incidence of acute GVHD (aGVHD), chronic GVHD (cGVHD), and GVHD-free relapse-free survival (GRFS). Neutrophil engraftment was identified as the first instance of an absolute neutrophil count over 0.5 × 10^9/L sustained for three consecutive days. Platelet engraftment was defined as the first occurrence of a platelet count exceeding 20 × 10^9/L for seven consecutive days without the need for transfusion support. The severity of aGVHD and cGVHD was graded according to established criteria, with cGVHD evaluation particularly performed in patients surviving beyond 100 days post-transplantation [14], [15]. GRFS refers to survival without grade III to IV aGVHD severe cGVHD, disease relapse, or death [16].

### Statistical analysis

The cutoff date was August 31, 2024. Kaplan-Meier survival curves were used to estimate OS, LFS, and GRFS, with group comparisons performed using the log-rank test. NRM, CIR, and GVHD were determined using the Fine-Gray method [17]. Relapse and NRM were treated as reciprocal competing risks, while relapse and death without GVHD were regarded as competing events for all GVHD assessments. Variables reaching a significance level of *P* < 0.20 in the univariate analysis were subjected to multivariate analyses. In multivariate analyses, the Cox proportional hazards regression model with backward stepwise regression was used to identify independent factors influencing OS, LFS and GRFS, while the Fine-Gray proportional hazards regression model was constructed for competing-risk endpoints. All P-values were two-sided, with a *P*-value of < 0.05 considered statistically significant. Statistical analyses were conducted using SPSS version 26.0 (IBM, Armonk, NY) and R version 4.2.2 (R Foundation for Statistical Computing, Vienna, Austria).

### Data availability statement

All data are available in the main text. Raw data can be obtained from the corresponding authors upon formal request.

## Results

### Patient characteristics

A total of 51 R/R B-ALL patients were enrolled in our cohort, with a median age of 32.1 years (range, 14.6–67.1). Detailed baseline characteristics and transplantation data are presented in Table1. Of these patients, 41.2 % (n = 21) were deemed as poor risk cytogenetics. Genetic abnormalities included BCR/ABL-positive in 10 patients (19.6 %), IKZF1 deletion/mutation in 9 (17.7 %), MLL-AF4 fusion in 3 (5.9 %), PAX5 mutation in 3 (5.9 %), ETV6 deletion/mutation in 3 (5.9 %), RUNX1 mutation in 2 (3.9 %), and TP53 mutation in 1 (2.0 %). A complex karyotype was identified in 8 cases (15.7 %). Thirty-nine patients (76.5 %) had relapsed B-ALL, and 12 (23.5 %) had refractory B-ALL. The median number of prior therapy lines was four (range, 2–24). One patient had previously undergone URD-HSCT, and two patients had previously received CAR-T therapy (one with universal CD19 CAR-T therapy and one with autologous CD19 CAR-T therapy). EMD involvement was detected in five individuals (9.8 %), including two cases of central nervous system leukemia. The median percentage of BM blasts at baseline was 41 % (range, 2–94 %).

**Table 1:** Baseline characteristics and transplantation.

**Characteristics**	**Patients (N = 51)**
**Gender, n (%)**	
Male	25 (49.0)
Female	26 (51.0)
**WBC count at diagnosis, median (range, ×10^9^/L)**	26.5 (1.6–497.4)
**Interval from diagnosis to CAR-T, median (range, month)**	6.5 (2.1–109.1)
**Age at HSCT**, **median** (**range**, **years**)	32.1 (14.6–67.1)
Risk stratification, n (%)*	
Good	30 (58.8)
Poor	21 (41.2)
**BCR/ABL1 transcripts at diagnosis, n (%)**	10 (19.6)
**Number of prior therapies, median (range)**	4 (2–24)
**Disease status at CAR-T, n (%)**	
Refractory	12 (23.5)
Relapse	39 (76.5)
**BM blasts at CAR-T enrolment, median (range, %)**	41 (2–94)
**EMD involvements, n (%)**	5 (9.8)
**Targets of CAR-T, n (%)**	
CD19	36 (70.6)
CD22	2 (3.9)
Dual CD19 and CD22	13 (25.5)
**CAR-T infusion dose, median (range, ×10^6^ cells/kg)**	2.7 (0.9–10.5)
**Interval from CAR-T to HSCT, median (range, months)**	2.6 (1.8–4.1)
**Disease status before HSCT, n (%)**	
CR1	12 (23.5)
CR2	28 (54.9)
≥CR3	11 (21.6)
**Conditioning regimens, n (%)**	
Myeloablative	48 (94.1)
Reduced-intensity	3 (5.9)
**Donor type, n (%)**	
Haploidentical-related donors	45 (88.2)
Unrelated donors	5 (9.8)
Matched sibling donors	1 (2.0)
**ABO compatibility, n (%)**	
Identical	22 (43.1)
Incompatibility	29 (56.9)
**Donor-recipient sex matched, n (%)**	
Identical	23 (45.1)
Mismatch	28 (54.9)
**MNCs dose, median (range, ×10^8^/kg)**	13.0 (3.0–35.4)
**CD34^+^ cell dose, median (range, ×10^6^/kg)**	5.6 (2.7–15.4)
**Neutrophil engraftment, median (range, days)**	13 (10–29)
**Platelet engraftment, median (range, days)**	15 (7–29)
**aGVHD, n (%)**	
I-IV	16 (31.4)
II-IV	8 (15.7)
**cGVHD, n (%)**	23 (45.1)
**Prophylactic DLI, n (%)**	21 (41.2)

aGVHD, acute graft-versus-host disease; BM, bone marrow; CAR-T, Chimeric antigen receptor-T; cGVHD, chronic graft-versus-host disease; CR, complete remission; DLI, donor lymphocyte infusion; EMD, extramedullary disease; HSCT, hematopoietic stem cell transplantation; MNCs, mononuclear cells; WBC, white blood cell.

* Poor risk are defined as the presence of adverse cytogenetic abnormalities including hypodiploidy, t(4;11), or t(1;19), other KMT2A rearrangements, BCR-ABL1–like or Ph-like ALL, complex karyotype, and iAMP21.

### CAR-T therapy as bridging to allo-HSCT

The detailed characteristics of the CAR-T product are summarized in Table S1. Median time from the initial diagnosis to CAR-T infusion was 6.5 months (range, 2.1–109.1). Thirty-six patients (70.6 %) received CD19-targeted CAR-T cells, 13 (25.5 %) received dual CD19-CD22 CAR-T cells, and 2 (3.9 %) received CD22-targeted CAR-T cells. The median dose of CAR-T cells administered was 2.7 × 10^6^ cells/kg (range, 0.9–10.5). All patients experienced cytokine release syndrome (CRS) after CAR-T cell infusion, including 23.5 % of patients with grade 1 CRS, 66.7 % with grade 2 CRS, and 9.8 % with grade 3 CRS. No cases of grade 4 or 5 CRS were reported. All CRS episodes were successfully managed following standard intervention with glucocorticoids only in 3 patients (5.9 %), tocilizumab in 6 patients (11.8 %), tocilizumab plus glucocorticoids in 17 patients (33.3 %), and symptomatic treatment in 25 patients (49.0 %).

**Fig. 1:**
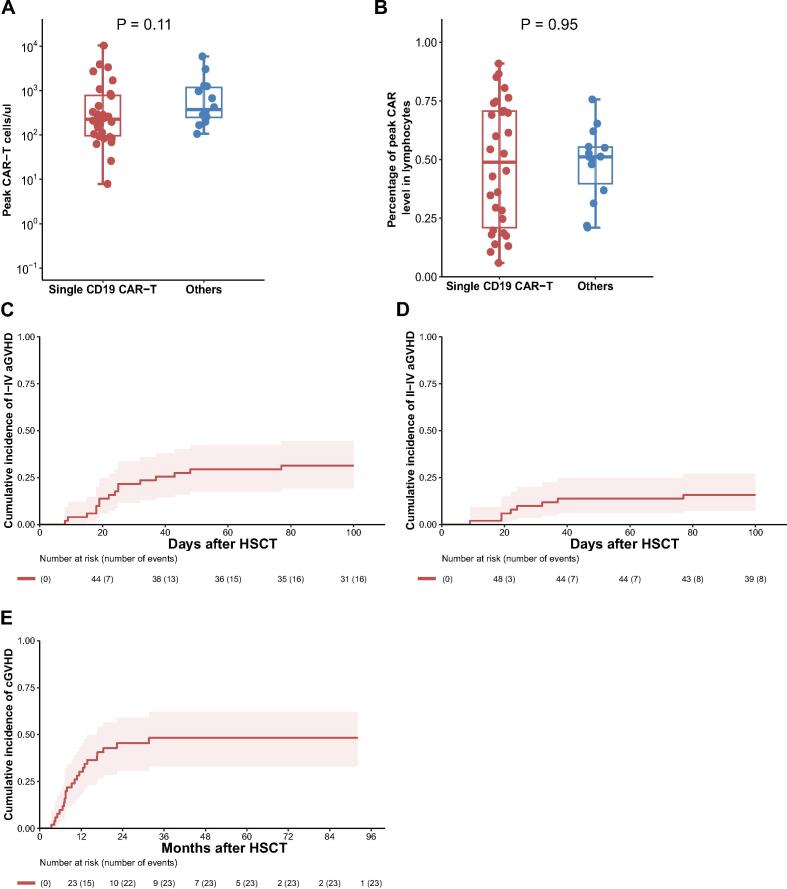
CAR-T cell dynamics and cumulative incidence of GVHD. (A) Comparison of peak CAR-T cell counts (cells/μL) between single CD19 CAR-T recipients and others (Dual CD19-CD22 CAR-T and single CD22 CAR-T). (B) Comparison of the percentage of peak CAR-T cells in lymphocytes between patients who received single CD19 CAR-T therapy and those who received other CAR-T therapies (Dual CD19-CD22 CAR-T and single CD22 CAR-T). (C) Cumulative incidence of grades I-IV acute GVHD (aGVHD) following HSCT. (D) Cumulative incidence of grades II-IV aGVHD following HSCT. (E) Cumulative incidence of chronic GVHD (cGVHD) over time post-HSCT.

Robust expansion of CAR-T cells in peripheral blood was observed in most patients, with a median peak proliferation level and percentage in lymphocytes of 270.5 cells/μL (range, 7.9–10,280) and 51.2 % (range, 5.9–91.0 %), respectively. Peak expansion levels and pecentage of CAR-T cells did not vary significantly between patients receiving single CD19-targeted CAR-T cells and those receiving dual CD19-CD22 or CD22-targeted CAR-T cells (*P* = 0.11 and *P* = 0.95) (Fig. 1A–B). Prior to transplantation, five patients had BCA recovery, while the remaining patients had persistent BCA. Median proportion of CAR-T cells in lymphocytes in BM before allo-HSCT was 2.3 % (range, 0.2–62.0 %).

All patients in MRD^-^ status underwent sequential allo-HSCT within 6 months following CAR-T infusion, with a median interval of 2.6 months (range, 1.8–4.1). Twelve patients (23.5 %) were transplanted in first CR, 28 (54.9 %) in second CR, and 11 (21.6 %) in third CR or beyond. Forty-eight patients (94.1 %) were preconditioned with the MAC regimen, whereas three (5.9 %) with the RIC regimen. Haplo-HSCT was the predominant transplant type, performed in 88.2 % of patients, while URD-HSCT and MSD-HSCT were performed in 9.8 % and 2.0 %, respectively. The median doses of infused mononuclear cells (MNCs) and CD34 + cells were 13.0 × 10^8^/kg (range, 3.0–35.4) and 5.6 × 10^6^/kg (range, 2.7–15.4), respectively.

### Post-HSCT engraftment, GVHD and other complications

Neutrophil engraftment occurred in all patients with a median time to engraftment of 13 days (range, 10–29). Platelet engraftment was successful in 96.0 % of patients at a median time to engraftment of 15 days (range, 7–29), with two patients experiencing platelet engraftment failure. aGVHD of all grades was observed in 16 (31.4 %) patients, with 8 patients being diagnosed with grade I aGVHD, 4 with grade II, 3 with grade III, and 1 with grade IV. Median time of aGVHD onset was 23 days (range, 8–77). The cumulative incidence of aGVHD at day 100 was 31.4 % (95 % CI, 19.2–44.3 %), with grades II-IV aGVHD occurring in 15.7 % (95 % CI, 7.27–27.0 %) (Fig. 1C–D). All patients were eligible for cGVHD evaluation, and 23 (45.1 %) developed cGVHD. The majority of cGVHD events (n = 15, 65.2 %) occurred within the first year, 7 (30.4 %) in the second year, and 1 (4.3 %) in the third year post-HSCT. The cumulative incidence of cGVHD was 30.2 % (95 % CI, 19.7–45.6 %) at 1 year, 45.4 % (95 % CI, 30.7–59.0 %) at 2 years and 48.3 % (95 % CI, 33.0–62.0 %) at 4 years (Fig. 1E).

Twelve patients (23.5 %) experienced clinical hemorrhagic cystitis. One instance of Epstein-Barr virus-associated post-transplant lymphoproliferative disorder occurred 1.8 months after transplantation, which responded rapidly to rituximab. One episode of immune thrombocytopenia developed 4.1 months post-transplant, and the patient achieved remission following intravenous high-dose immunoglobulin therapy. Two patients had transplant-associated microangiopathy (TAM). Other organ toxicities included cataracts (n = 3, 5.9 %), nephrotic syndrome (n = 2, 3.9 %), femoral head necrosis (n = 2, 3.9 %), and heart failure (n = 1, 2.0 %).

### Survival outcomes

**Fig. 2:**
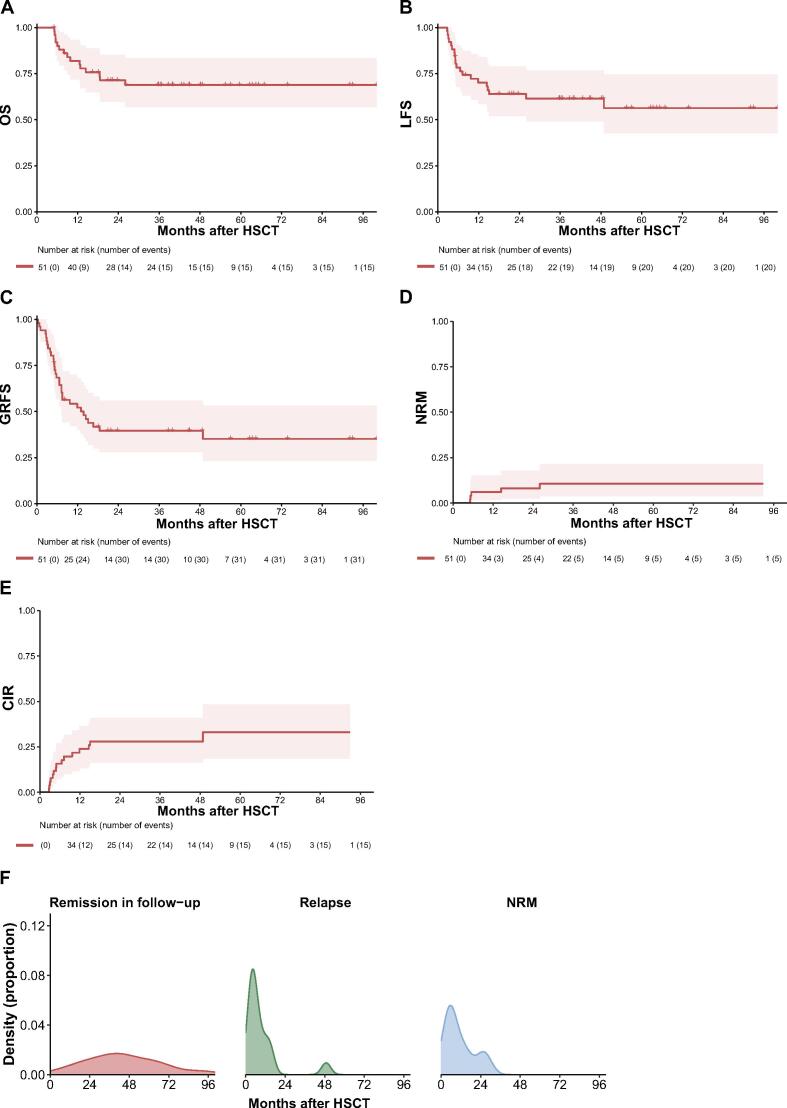
Long-term outcomes of OS (A), LFS (B), GRFS (C), NRM (D) and CIR (E). (F) Density plot with incidence from time since allo-HSCT. Left panel, patients in remission with last known follow-up indicated. Middle panel, incidence of leukemia relapse marked at the time of relapse after allo-HSCT. Right panel, incidence of deaths due to NRM.

A total of 21 patients (41.2 %) received prophylactic DLI after allo-HSCT. At a median follow-up duration of 43.2 months (range, 5.0–100.0) for the surviving patients, 16 deaths occurred, with disease relapse being the most frequent cause (n = 10), followed by respiratory failure (n = 3), severe infections (n = 2), and heart failure (n = 1). The estimated probabilities of 2-year and 4-year OS rates for the entire cohort were 71.4 % (95 % CI, 59.8–85.3 %) and 68.9 % (95 % CI, 56.9–83.4 %), respectively, while LFS rates at 2 years and 4 years were 64.0 % (95 % CI, 51.9–78.8 %) and 61.4 % (95 % CI, 49.1–76.8 %), respectively (Fig. 2A–B). The corresponding rates of GRFS at 2 years and 4 years were both 39.5 % (95 % CI, 28.0–55.9 %) (Fig. 2C).

Relapse was confirmed in 15 patients (29.4 %), with the majority (n = 11) experiencing antigen-positive relapse (Table S2). Among these, 13 had hematologic relapses, 1 had isolated extramedullary relapse, and 1 had combined hematologic and extramedullary relapses. The median time to relapse was 5.0 months (range, 2.7–48.9) post-transplant, with 80 % of relapses (n = 12) occurring within the first year, 13.3 % (n = 2) in the second year, and 6.7 % (n = 1) at over 4 years. The median time to relapse was 4.9 months (range, 2.7–48.9) for patients with antigen-positive relapse and 8.1 months (range, 4.1–15.1) for those with antigen-negative relapse. Nine patients (60 %) succumbed to disease relapse, and 1 (6.7 %) to severe infection. NRM at 2 years was 8.1 % (95 % CI, 2.5–17.9 %), and at 4 years was 10.6 % (95 % CI, 3.8–21.6 %). CIR was 28.0 % (95 % CI, 16.3–40.9 %) at both 2 years and 4 years (Fig. 2D–F).

### Univariate and multivariate analysis

**Fig. 3:**
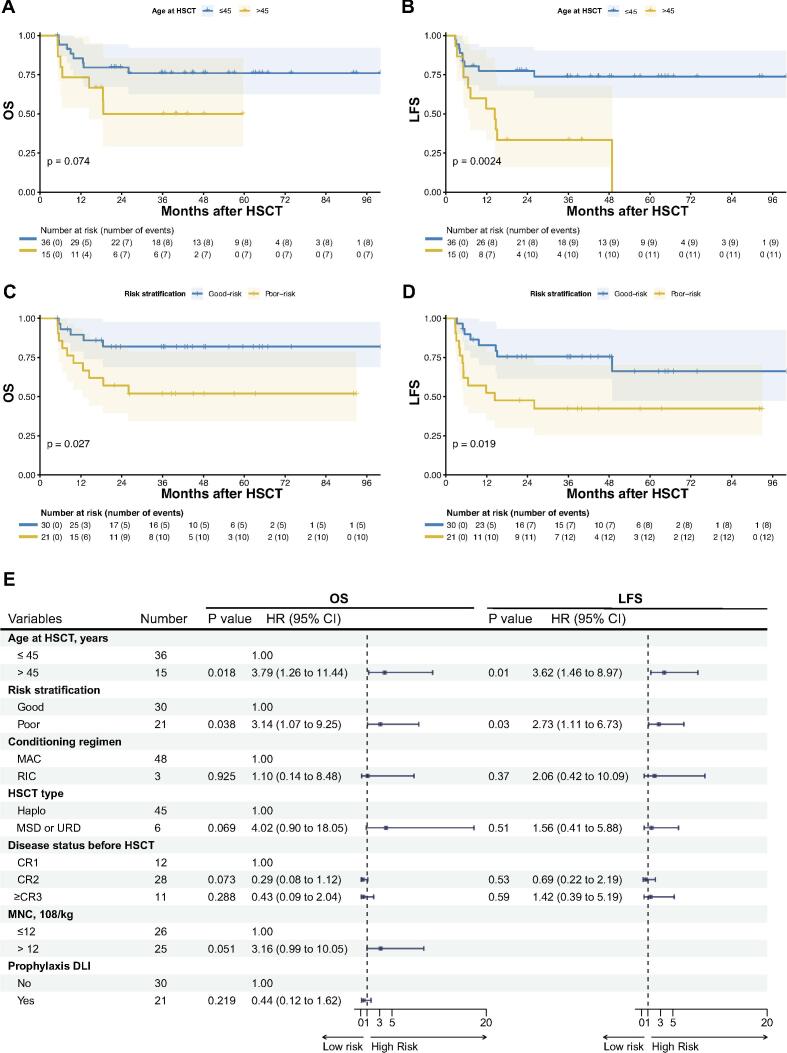
Impact of key clinical factors on survival outcomes following allo-HSCT. (A–B) Univariate survival analyses of OS (A) and LFS (B) by age. (C–D) Univariate survival analyses of OS (C) and LFS (D) by risk stratification. (E) Forest plot for multivariable analysis of OS and LFS.

Univariate analyses were conducted to identify baseline variables associated with clinical outcomes. We found that older age at allo-HSCT (>45 years) was associated with poor OS (*P* = 0.074) and LFS (*P* = 0.002) (Fig. 3A–B). Poor-risk stratification was linked to worse OS (*P* = 0.027) and LFS (*P* = 0.019) (Fig. 3C–D). Other factors including the type of conditioning regimen (*P* = 0.149; *P* < 0.001), type of allo-HSCT (*P* = 0.064; *P* = 0.199), and disease status before allo-HSCT (*P* = 0.147; *P* = 0.096), were included in the multivariate analysis for OS and LFS. Additionally, MNCs dose (*P* = 0.132) and the use of prophylactic DLI (*P* = 0.175) were also included in the multivariate analysis for OS. For GRFS, both the type of conditioning regimen (*P* = 0.053) and age at allo-HSCT (*P* = 0.052) were considered for multivariate analysis. Variables influencing CIR included age at allo-HSCT (*P* = 0.027), patient gender (*P* = 0.134), risk stratification (*P* = 0.201), type of conditioning regimen (*P* = 0.026), and the use of prophylactic DLI (*P* = 0.151). In terms of aGVHD, age at allo-HSCT (*P* = 0.070) and the target of CAR-T (*P* = 0.143) were included. Univariate analysis for grade II-IV aGVHD identified that only donor-recipient ABO mismatch was suggested as a poor prognostic factor (*P* = 0.007). For cGVHD, the type of conditioning regimen (*P* = 0.003) and MNCs dose (*P* = 0.152) were further analyzed. White blood cell count at diagnosis, BCR/ABL status, the interval between CAR-T and allo-HSCT, donor-recipient gender match, and CD34 + cell dose were not associated with either clinical outcome.

**Fig. 4:**
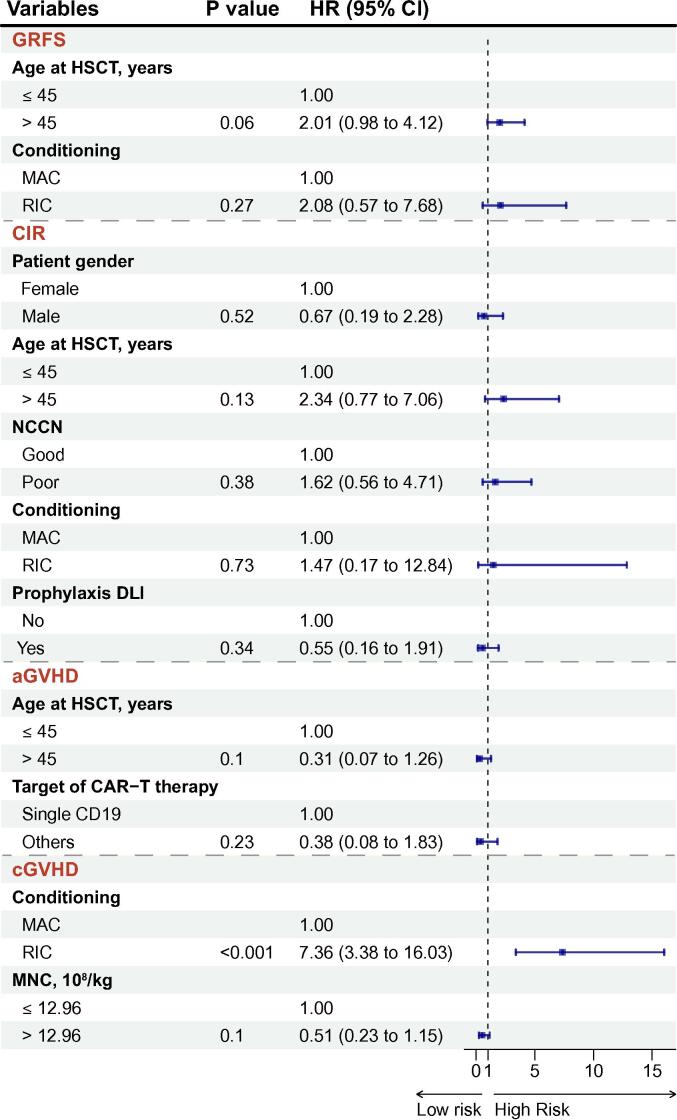
Forest plot for multivariable analysis of GRFS, CIR, aGVHD, and cGVHD.

Multivariate analyses for OS and LFS confirmed that advanced age at transplant negatively affected OS (hazard ratio [HR]: 3.79, 95 % confidence interval [CI]: 1.26–11.44, *P* = 0.018) and LFS (HR: 3.62, 95 % CI: 1.46–8.97, *P* = 0.01) (Fig. 3E). Poor-risk NCCN stratification also strongly contributed to worse OS (HR = 3.14, 95 % CI: 1.07–9.25, *P* = 0.038) and LFS (HR = 2.73, 95 % CI: 1.11–6.73, *P* = 0.03). No remarkable independent factors were identified in association with GRFS, CIR, or aGVHD. In the multivariate analysis of cGVHD, the use of RIC regimen emerged as a significant risk factor for the development of cGVHD (HR: 7.36, 95 % CI: 3.38–16.03, *P* < 0.001) (Fig. 4).

## Discussion

Unequivocally, MRD^-^CR status is advantageous for sequential transplantation to consolidate the therapeutic effect and reduce relapse risk. However, not all patients may benefit equally from allo-HSCT owing to potential transplant-related complications. We previously reported the initial results of this sequential therapy in the pre-transplant MRD^-^ cohort, with superior outcomes of 2-year OS and LFS in the bridging haplo-HSCT group compared to the CAR-T alone group (83.3 % vs. 36.4 % and 76.1 % vs. 32.8 %), respectively [7]. A separate clinical trial conducted in China also found that patients who received allo-HSCT after CAR-T therapy had significantly better LFS (76.9 % vs. 11.6 %) and OS (79.1 % vs. 32.0 %) compared to those receiving CAR-T alone [5]. These findings are in line with results by Hay et al. and Jiang et al. that consolidative allo-HSCT significantly improves survival outcomes in MRD^-^CR patients following CAR-T therapy [6], [18]. Conversely, a study by Memorial Sloan Kettering Cancer Center demonstrated, which demonstrated that bridging transplantation did not provide significant additional benefits in the MRD- CR population. This could be attributed to several factors, including the persistence of CAR-T cells, the high toxicity associated with HSCT, and the absence of multivariate analysis in their study design, which may have obscured potential differences in outcomes [4]. Here, we retrospectively analyzed long-term outcomes in a larger MRD^-^CR cohort. Our results corroborate these previous findings, demonstrating that patients achieving MRD^-^CR had favorable outcomes following allo-HSCT, with 2-year OS and LFS of 71.4 % and 64.0 %, respectively. With extended follow-up, sequential allo-HSCT continued to contribute to durable remission and survival benefits in this patient cohort, with 4-year OS and LFS of 68.9 % and 61.4 %, comparable to the long-term outcomes observed with conventional allo-HSCT following chemotherapy. Notably, our study recorded the longest survival period at 100 months, highlighting the potential long-term advantages of this approach.

Despite these promising outcomes, our findings also underscore considerable relapse risks with 2-year CIR of 28.0 %. Antigen-positive relapse predominated, occurring mostly within the first year post-HSCT. This early relapse pattern underscores the limits of allo-HSCT in completely eradicating residual disease, even in individuals who achieve profound molecular response prior to allo-HSCT. Furthermore, four instances of CD19-negative relapse were also observed at later time points, suggesting the existence of an antigen-negative leukemic population probably resistant to both CD19 CAR-T therapy and allo-HSCT. The prognosis for patients who relapse post-HSCT was particularly poor, with most patients in our cohort succumbing to disease progression. These findings highlight the critical need for innovative post-transplant strategies to prevent post-transplant relapse, particularly in high-risk populations.

The safety of sequential therapy has been a major focus of concern. In sequential CAR-T and allo-HSCT, the reported incidence of aGVHD varies from 23.3 % to 73.7 %, with grades II-IV aGVHD occurring in 0–21 % of cases, and cGVHD rates ranging from 21 % to 88.9 % [7], [19], [20], [21], [22], [23], [24]. In our research, we observed comparable incidences of aGVHD, with grades I-IV aGVHD incidence at 31.4 % and grades II-IV aGVHD at 15.7 %. The incidence of cGVHD at 2- and 4-years post-transplant was 45.4 % and 48.3 %, respectively. Importantly, none of the individuals died from GVHD. The NRM rate was very low, with fatalities mostly due to infections and organ failure. Additionally, the sequential treatment had no effect on neutrophil or platelet engraftment. These findings suggest that, in the long term, successive allo-HSCT following CAR-T cell infusion does not increase the risk of transplant-related complications or mortality, reinforcing the safety of this sequential therapeutic approach.

In this study, age and risk stratification were identified as significant independent risk factors influencing both OS and LFS. Age is a critical factor in transplant outcomes, with younger patients generally faring better due to a combination of factors, including fewer comorbidities, better overall health, and a more robust immune system capable of handling the stress of transplantation and subsequent GVHD. Our findings are consistent with several studies that have highlighted age-related disparities in post-transplant survival rates, with younger patients demonstrating superior OS and LFS [25], [26], [27]. On the other hand, we further confirmed that higher-risk disease, characterized by adverse cytogenetics, had a deleterious influence on long-term survival outcomes. This shows that even sequential therapy might be inadequate to improve the inferior outcomes associated with genomic instability. Additional therapeutic strategies or closer monitoring are warranted to improve their chances of long-term survival in this high-risk population. Collectively, while the combination of CAR-T therapy with allo-HSCT provides considerable advantages, the impact of age and disease risk stratification on outcomes indicates that treatment regimens must be tailored to the specific needs and characteristics of each patient.

While our study offers valuable insights, its retrospective design and the relatively small sample size from a single center pose limitations. Larger prospective multi-center studies are needed to fully understand the long-term efficacy and safety of the sequential approach. Second, our study’s single-arm design precludes direct comparisons between outcomes in MRD^-^ patients who did and did not undergo bridging allo-HSCT. Therefore, the observed benefits of allo-HSCT in our cohort should be interpreted with caution. Furthermore, although our cohort predominantly consisted of patients who underwent haplo-HSCT (88.2 %), the clinical outcomes are comparable to those observed in studies involving consolidative transplants mainly with MSD or URD [18], [21]. While we recognize that the emphasis on haplo-HSCT may limit the generalizability of our findings to other transplant groups, we believe that the results still offer valuable insights, particularly in settings where haplo-HSCT is commonly used.

## Conclusion

Collectively, our study demonstrates that allo-HSCT following CAR-T therapy in MRD^-^ R/R B-ALL patients can lead to durable remissions and meaningful survival benefits. However, the significant risk of relapse, especially in high-risk groups, necessitates tailored strategies and ongoing monitoring. The findings of this study have substantial therapeutic implications for managing patients with R/R B-ALL following CAR-T therapy.

## Patient consent for publication

Due to the retrospective nature of the study, the requirement for written informed consent was waived. All data used in this manuscript were anonymized to ensure patient confidentiality.

## Compliance with ethics requirement

This study involves human participants and was approved by the Ethics Review Committee of the First Affiliated Hospital of Zhejiang University (No.2020-416-2, No.2020-401, No.2019-1537, No.2018-747-2 and No.2018-747-5). Participants provided informed consent before taking part in the study.

## Author contributions

T.Y. and Y.D. contributed to the manuscript through data curation, formal analysis, and writing - original draft preparation. J.S., M.Z., J.F., S.F., P.X., R.H., Y.L., Y.Z., J.Y., X.L., L.L., H.F., Y.Y., G.W. and Y.H. contributed through investigation by enrolling and treating patients, as well as gathering data. D.K. contributed through formal analysis with a focus on statistical analysis. H.X. and A.H.C. were responsible for resources through CAR-T product manufacturing. J.C. and S.H. performed all flow cytometric analysis related to CAR T- cell detection. H.H. and Y.H. contributed through supervision and provided project administration, as well as gave final approval of the manuscript. All authors reviewed the content and approved the submitted version.

## Declaration of competing interest

The authors declare that they have no known competing financial interests or personal relationships that could have appeared to influence the work reported in this paper.
