# Supplementary material for: Sequential allogeneic HSCT after CAR-T therapy for relapsed/refractory acute lymphoblastic leukemia patients: A long-term follow-up result
Source: J Adv Res. 2025 Feb 11;78:461–9. doi: 10.1016/j.jare.2025.02.006 (PMC12684954; doi:10.1016/j.jare.2025.02.006)
Supplement: Supplementary Data 1 [file mmc1.docx]

Table S1. CAR-T product characteristics.

| **Characteristics** | **Value (N=51)** |
| --- | --- |
| CAR-T cell manufacture, median (range, days) | 9 (7-15) |
| CAR-T cell dose, median (range, 10^6^ cells/kg) | 2.7 (0.9-10.5) |
| CAR-T cell viability, median (range, %) | 92.1 (87.0-97.1) |
| CAR transduction rate, median (range, %) | 58.3 (18.2-92.6) |
| CAR-T cell target, n (%) |  |
| CD19 | 36 (70.6) |
| CD22 | 2 (3.9) |
| Dual CD19 and CD22 | 13 (25.5) |
| CAR-T cell source, n (%) |  |
| Murine CD19 | 11(21.6) |
| Humanized CD19 | 25 (49.0) |
| Humanized CD22 | 2 (3.9) |
| Murine CD19+ Humanized CD22 | 8 (15.7) |
| Humanized CD19+ Humanized CD22 | 5 (9.8) |
| CAR-T cell preparation, n (%) |  |
| Fresh | 30 (58.8) |
| Frozen | 21 (41.2) |

Table S2. Characteristics of relapsed patients.

| **Pt** | **Prior** EMD **involvement** | **Relapse type** | **Blasts (%) in BM at relapse** | **MRD (%) in BM at relapse** | **Antigen relapse type** | **Immunophenotype at baseline** | **Immunophenotype at relapse** | **OS/LFS (months)** |
| --- | --- | --- | --- | --- | --- | --- | --- | --- |
| 3 | No | BM | 82 | 83.8 | Positive | CD45^dim^CD34^+^CD38^+^CD19^+^CD22^+^CD20^+^CD24^+^CD10^-^ | CD45^dim^CD34^+^CD38^+^CD19^+^CD22^+^CD20^+^CD24^+^CD10^-^ | 9.0/5.0 |
| 4 | No | BM | 28 | 8.8 | Negative | CD45^+^CD34^+^CD38^+^CD19^+^CD22^+^CD20^+^ | CD45^dim^CD34^part^CD38^+^CD19^-^CD22^-^CD20^-^CD13^+^HLA-DR^+^ | 9.8/4.1 |
| 6 | No | BM | 68 | 64.8 | Positive | CD45^dim^CD34^+^CD38^-^CD19^+^CD20^-^HLA-DR^+^  CD101^+^CD10^+^CD13^-^ | CD45^dim^CD34^part^CD38^-^CD19^+^CD20^+^HLA-DR^+^CD10^+^ | 48.1/11.9* |
| 12 | No | BM | 29 | 26.4 | Positive | CD45^dim^CD34^+^CD33^+^CD38^-^CD19^+^CD22^+^CD20^+^CD10^+^  cyCD79a^+^TdT^+^ | CD45^dim^CD34^+^CD33^+^CD38^-^CD19^+^CD22^+^CD20^+^CD10^+^ | 12.6/3.9 |
| 13 | No | BM | 44 | 49.0 | Positive | CD45^dim^CD34⁺CD33⁺CD38^+^CD19⁺CD22⁺CD20⁺CD24⁺  CD200⁺CD10⁺ | CD45^dim^CD34⁺CD38^dim^CD19⁺CD22⁺CD20^part^CD58⁻CD81⁻  CD10^part^ | 59.6/48.9* |
| 14 | No | BM | 73 | 65.0 | Positive | CD45^dim^CD34⁺CD38^dim^CD19^+^CD22^+^CD20⁻CD10⁺ | CD45^dim^CD34⁺CD38⁺CD19⁺CD22^dim^CD20⁻CD58⁺CD81⁺  CD10⁺ | 16.4/7.2* |
| 17 | No | EMD | / | / | Positive | CD45^dim^CD34⁻CD38⁺CD19^+^CD22^dim^CD20⁻CD58^dim^CD81⁺CD10^part^ | TDT⁺PAX-5⁺CD19⁺ CD22⁺ CD20⁻ (Immunohistochemistry for extramedullary disease) | 43.2/3.3* |
| 19 | No | BM | 65 | 46.1 | Positive | CD45^dim^CD34⁺CD38^dim^CD19⁺CD22^dim^CD20^dim^CD58^dim^  CD81^dim^CD10⁺ | CD45^dim^CD34^+^CD38^dim^CD19^+^CD22^+^CD20^-^ CD58^+^ CD81^dim^  CD10^+^ | 8/3.1 |
| 25 | No | BM | 97 | 75.5 | Negative | CD45^dim^CD34^part^CD38⁺CD19⁺CD22⁺CD20⁻CD58⁺CD81⁻  CD10⁺ | CD45^dim^CD34^part^CD38⁺CD19⁻CD22⁺CD20⁻CD58⁺CD81⁻  CD10⁺ | 6/15.1 |
| 27 | No | BM | 94 | 83.2 | Negative | CD45^dim^CD34⁺CD33⁺CD38^-^CD19⁺CD22⁺CD20^+^CD200⁺ cyCD79a⁺TdT⁺CD58^-^CD81^-^CD10⁺ | CD45^dim^CD34^part^CD38^-^CD19^-^CD22^-^CD20^part^CD58^-^CD81^-^CD10^+^ | 18.6/6.6 |
| 30 | No | BM | 25 | 43.9 | Positive | CD45^dim^CD38^+^CD19^+^CD22^+^CD20^+^cyCD79a^+^CD10^-^ | CD45^dim^CD38^+^CD19^+^CD22^+^CD20^+^CD200^+^cyCD79a^+^CD10^-^ | 5.5/2.7 |
| 32 | No | BM | 74 | 73 | Positive | CD45^dim^CD34^part^CD38^+^CD19^+^CyCD79a^+^CD15^+^CD10^part^  HLA-DR^+^ | CD45^dim^CD34^part^CD38^+^CD19^+^CD22^+^CD20^+^HLA-DR^+^  CyCD79a^+^CD15^+^CD10^-^ | 18.5/14.7 |
| 39 | No | BM | 7 | 0.9 | Negative | CD45^dim^CD34^part^CD38⁻CD19⁺CD22^dim^CD20⁻CD58^dim^CD81⁺CD10⁻ | CD45^dim^CD34^part^CD38⁺CD19⁻CD22⁺CD20⁻CD58⁺CD81^dim^ CD10⁻ | 12.8/9.7 |
| 41 | No | BM and EMD | 80 | 76.4 | Positive | CD45^dim^CD34⁻CD38⁺CD19⁺CD22⁺CD20⁻CD58⁺CD81^part^  CD10⁺ | CD45^dim^CD38^+^CD19^+^CD22^+^CD20⁻CD200^+^cyCD79a^+^nTdT^+^  CD10^+^ | 26.2/4.9* |
| 49 | No | BM | 8 | 20.1 | Positive | CD45^dim^CD34⁻CD38⁺CD19⁺CD22⁺CD20^+^CD10⁺ | CD45^dim^CD34⁻CD38⁺CD19⁺CD22⁺CD20^+^CD10⁺ | 6.5/2.9 |

*Ongoing follow-up

BM, bone marrow; EMD, extramedullary disease; LFS, leukemia-free survival; MRD, minimal residual disease; OS, overall survival; Pt, patient.
